# A phenol/chloroform-free method to extract nucleic acids from recalcitrant, woody tropical species for gene expression and sequencing

**DOI:** 10.1186/s13007-019-0447-3

**Published:** 2019-06-05

**Authors:** François F. Barbier, Tinashe G. Chabikwa, Muhammad U. Ahsan, Stacey E. Cook, Rosanna Powell, Milos Tanurdzic, Christine A. Beveridge

**Affiliations:** School of Biological Sciences, and The Queensland Alliance for Agriculture and Food Innovation, The University of Queensland, St. Lucia, QLD 4072 Australia

**Keywords:** DNA RNA extraction, CTAB, SDS, Woody species, Macadamia, Avocado, Mango

## Abstract

**Background:**

Woody tropical plants contain high levels of complex organic compounds that inhibit the chemical procedures needed to extract RNA or DNA, thus compromising downstream applications such as RNA sequencing and analysis of gene expression. To overcome this issue, researchers must use extraction protocols using CTAB/PVP buffer instead of commercially available DNA/RNA extraction kits. However, these protocols are time-consuming, use toxic chemicals like phenol and chloroform, and can only be used to process a small number of samples at a time. To overcome these issues, we developed a new CTAB/PVP based protocol for RNA or DNA extraction that eliminates the traditional phenol/chloroform step. Furthermore, the protocol was developed for 96-well plates to speed up processing.

**Results:**

Our new protocol enabled us to successfully extract RNA from macadamia, avocado, and mango tissues that are traditionally difficult to work with. This RNA was then successfully used to synthesise cDNA for real-time quantitative PCR and to generate good quality RNA-Seq libraries. Our protocol can be easily converted for rapid DNA extraction from different tropical and sub-tropical tree species.

**Conclusion:**

This method enables safer and faster DNA and RNA extraction from recalcitrant species, thus facilitating future work on tropical trees.

**Electronic supplementary material:**

The online version of this article (10.1186/s13007-019-0447-3) contains supplementary material, which is available to authorized users.

## Background

Molecular experiments reveal the physio-genetic structure and function of plant species, enabling growers to improve productivity in changing environmental conditions. However, the protocols used to extract RNA or DNA from plants were developed using herbaceous species such as Arabidopsis (*Arabidopsis thaliana* L. (Heyn)) [1–4], and do not work well for some taxa. For example, the tissues of tropical trees contain polysaccharides and polyphenols that compromise the extraction of nucleic acids [5, 6]. Extraction of RNA or DNA from these species relies on the use of phenol and chloroform, which are volatile, toxic, and therefore impractical for routine and repeated use by researchers. Therefore, we sought to improve the extraction of RNA or DNA from tropical trees by creating a protocol that is safer and faster.

For herbaceous plants, the extraction of RNA or DNA can be easily achieved with silica membranes, however these methods do not work efficiently with tropical woody species. Furthermore, this technique only retains RNA strands longer than fifty nucleotides, eliminating the small RNA which are of emerging importance in molecular plant sciences [7]. Therefore, in the current study we began with modifications to a protocol for RNA extraction from pine tree [8], which uses cetyl trimethylammonium bromide (CTAB) and polyvinylpolypyrrolidone (PVP) in the lysis buffer [2, 9–12]. CTAB is a cationic detergent that emancipates the contents of the inner cell and promotes the separation of proteins and polysaccharides from nucleic acids [13, 14]. PVP is a polyphenol oxidase inhibitor that inhibits the oxidation of the samples (browning), which often compromises nucleic acid quality and quantity [15, 16]. Sodium chloride is usually added at high concentrations to prevent the formation of CTAB-nucleic acid complexes [17], and to create an environment in which nucleic acids can precipitate but polysaccharides remain soluble [18]. Some protocols use Sodium Docecyl Sulfate-based (SDS) buffers for the lysis step. SDS separates proteins from nucleic acids but cannot prevent oxidation, thereby inhibiting downstream use of the nucleic acids. The downstream side of using the CTAB/PVP or SDS buffers to lyse the samples is that, not only are these protocols lengthy and low-throughput, but they require to use phenol and chloroform [5, 8, 9, 19, 20], which are volatile and toxic. Chloroform is even classified as “reasonably anticipated to be a human carcinogen based on sufficient evidence of carcinogenicity from studies in experimental animals” according the 14^th^ Report on Carcinogens from the U.S. Department of Health and Human Services [21]. Given the growing need of RNA extractions for large-scale transcriptomic studies, the use of such chemicals is not acceptable for routine extraction protocols [22].

In this study, we developed and tested a new method for extracting DNA and total RNA, including small RNA, from diverse tissues of tropical/subtropical woody species, including Avocado (*Persea americana* L.), Macadamia (*Macadamia integrifolia* L.) and Mango (*Mangifera indica* L.). Our method does not require phenol or cholorophorm and can be used to extract 96 samples in a 96-well plate in less than 1 day. The resultant RNA can be sequenced and used to produce good quality RNA-Seq reads. The DNA extraction method can also be used on Coffee tree (*Coffea arabica* L.) and Eucalyptus (*Eucalyptus grandis* L.) samples. This protocol will facilitate novel molecular research on tropical trees that have previously been too difficult for large-scale genetic survey and experimentation.

## Materials and methods

### Plant material and tissue grinding

Various tissue samples were collected from field-grown Avocado cv. *Hass*, Mango cv. *1243*, Macadamia cv. *751*, Coffee and Eucalyptus in Queensland, Australia. All the trees were mature (8–15 year old). DNA was extracted from mature leaves whilst RNA extractions were performed on stem, leaf, root, flower and axillary bud tissues. Arabidopsis cv. *Columbia*-*0* and garden pea cv. *Torsdag* were also used to assess the efficiency of the method on herbaceous plants.

Fresh material was flash frozen in liquid nitrogen or dry ice and stored at − 80 °C before being ground to powder (homogenised). Frozen samples were ground with an automated tissue grinder (Geno/Grinder^®^, SPEX). Leaf, bud, flower and root samples were ground in 2 ml tubes (up to 96 at a time) and stem tissues were ground in 15 ml vials (up to 12 at a time). Frozen samples were disrupted for 1 min at 1750 rpm and refrozen for 30 min at − 80 °C before being disrupted again for one or two cycles. The amount of powder required for the extraction was transferred in a new tube using a spatula. The estimated weight was assessed based on preliminary assessment volume/weight.

### Solutions and reagents

- CTAB buffer: CTAB 2%, NaCl 1.4 M, EDTA 20 mM, Tris–HCl 100 mM, PVP40 2%
- Isopropanol 100%
- DTT (DL-Dithiothreitol) 0.5 mM
- SDS 10%
- 70% ethanol
- DNase I + DNase buffer
- RNase A

### RNA extraction

#### Lysis of Tissues

- Transfer 0.5–50 mg (not more) of ground sample into a 2 ml tube
- Add 625 µl of CTAB buffer and 25 µl of DTT 0.5 mM (mix them prior to use)
- Vortex well and incubate at 60 °C for 15 min. Vortex every 5 min
- ONLY FOR RECALCITRANT SAMPLES: Add 65 µl of SDS 10% and vortex well (if you use small amount of tissue, only add 40 µl of SDS 10%)
  - Note: At that stage, SDS precipitates with CTAB making the sample cloudy
- Centrifuge at 20,000*g* for 15 min at room temperature
  - Note: Tissue debris will pellet in the bottom of the tube and SDS/CTAB will form a semi-solid layer at the surface
- Remove the tubes carefully from the centrifuge and transfer 550–600 µl of the liquid phase into a new 2 ml tube or into a 2 ml cavity of a 96 well plate and proceed to the nucleic acid precipitation
  - Note 1: In case too much top layer has been mistakenly taken up, put the samples into new 2 ml tubes and repeat this step (tip: after this second centrifuge, take the sample by pipetting directly from the bottom of the tube (the top layer will stick to the outside of the tip).
  - Note 2: Unclear precipitate can form depending on the tissue (this was the case with avocado buds). Transfer into a filter column (Qiagen QIAshredder or Macherey–Nagel NucleoSpin Filters) instead of the 2 ml tube and repeat this step.

#### Nucleic acid precipitation

- Add 550 µl of pre-cooled (− 20 °C) isopropanol into each tube/well and vortex well
- Leave at − 20 °C for 15 min
  - Note: At that stage you can leave the samples for days or weeks at − 20 °C
- Centrifuge the tubes/plate at 4 °C for 1 h at 3100*g* for plates or 45 min at 20,000*g* for tubes
- Tip the tubes/plate to remove isopropanol
- Add 1 ml 70% ethanol
- Centrifuge at 4 °C for 10–15 min at 3100*g* for plates or 20,000*g* for tubes
- Tip the tubes/plate to remove the ethanol
- Centrifuge the tubes/plate briefly and remove remaining ethanol with a pipette
- Let the samples dry on the bench for 10 min.

#### DNase treatment

- Add 175 µl of RNase-free water and pipette up and down to resuspend the pellet
- Place the plate at 50–60 °C for 2–3 min, vortex and quick spin down
- Add 20 µl of DNase buffer and 5 µl of DNase I per tube or well (mix them before)
- Vortex briefly, quick spin down and incubate at 37 °C for 20–30 min
- Immediately proceed to the next step.

#### RNA precipitation and elution

- Add 200 µl of pre-cooled (− 20 °C) isopropanol into each tube/well and vortex well
- Leave for 15 min at − 20 °C
  - Note: At that stage, you can leave the samples for several days at − 20 °C
- Centrifuge at 4 °C for 1 h at 3100 g for plates or 45 min at 20,000*g* for tubes
- Tip the tubes/plate to remove isopropanol
- Add 1 ml of 70% ethanol
- Centrifuge at 4 °C for 10–15 min at 3100 g for plates or 20,000*g* for tubes
- Tip tubes/plate to remove ethanol
- Centrifuge the tubes/plate briefly and remove remaining ethanol with a pipette
- Let the samples dry on the bench for 10 min
- Add 40–100 µl of warm (60 °C) RNase-free water and pipette up and down vigorously
- Vortex well and quick spin down
- Determine RNA quantity and quality by gel electrophoresis and spectrophotometry, and store the samples at − 80 °C.

### DNA extraction

#### Lysis of Tissues and RNase treatment

- Transfer up to 50 mg (no more) of ground sample to a 2 ml tube
- Add 400 µl of CTAB buffer and 4 µl of RNase A + RNase buffer (mix them together as before)
- Vortex well and incubate at 37 °C for 1 h. Vortex them and invert every 15 min
- Add 30 µl of SDS 10% and vortex well (if you use a small amount of tissue (< 10–15 mg), add only 15 µl of SDS 10%)
  - Note: SDS precipitates with CTAB making the sample cloudy
- Centrifuge at 20,000*g* for 15 min at room temperature
  - Note: Tissue debris will pellet in the bottom of the tube and SDS/CTAB will form a semi-solid layer at the surface
- Remove carefully from the centrifuge and transfer 350–400 µl of the liquid phase into a new 2 ml tube or into a 2 ml 96 well plate and proceed to the nucleic acid precipitation
  - Note 1: In case too much top layer has been mistakenly taken up, put the samples into new 2 ml tubes and repeat this step (tip: after this second centrifuge, take the sample by pipetting directly from the bottom of the tube (the top layer will stick to the outside of the tip).
  - Note 2: In case of dirty/unclear precipitate can form depending on the tissue (this was the case with avocado buds), transfer into a filter column (Qiagen QIAshredder or Macherey–Nagel NucleoSpin Filters) instead of the 2 ml tube and repeat this step.

#### DNA precipitation and elution

- Add 350–400 µl of pre-cooled (− 20 °C) isopropanol to each tube/well and vortex well
- Leave at − 20 °C for 15 min
  - Note: At that stage, you can leave the samples for several days at − 20 °C
- Centrifuge the tubes/plate at 4 °C for 1 h at 3100*g* for plates or 45 min at 20,000*g* for tubes
- Tip the tubes/plate to remove isopropanol
- Add 1 ml of 70% ethanol
- Centrifuge at 4 °C for 10–15 min at 3100*g* for plates or 20,000*g* for tubes
- Tip the tubes/plate to remove ethanol
- Centrifuge the tubes/plate briefly and remove remaining ethanol with a pipette
- Let the samples dry on the bench for 10 min
- Add 90 µl of warm (60 °C) RNase-free water, pipette up and down and vortex well to resuspend the pellet.

### Quantification and quality control

Quality control was performed by running RNA or DNA samples on a 1.1% agarose gel. The nucleic acids were stained by adding Red Sage^®^ to the gel. Gel visualisation was performed using the Gel Doc™ Gel Documentation System (Bio-Rad Laboratories, California, USA). The quality and purity of the samples were determined with a NanoVue™ Plus Spectrophotometer (GE Healthcare Life Sciences, Pennsylvania, USA) measuring the absorbance at 280 nm for RNA and 260 for DNA.

### cDNA synthesis and quantitative real-time PCR (qRT-PCR)

For qRT-PCR, cDNA was obtained by reverse transcription using 250–500 ng of total RNA in 8 µl and 2 µl of 5 × iScript Supermix (Bio-Rad Laboratories, California, USA), following the manufacturer’s instructions. The cDNA was then diluted to 0.5 ng equivalent RNA in milliQ water per µl for a working template solution. Quantitative real-time PCR was then performed using 2 µl of 1 mM primer mix, 5 µl of cDNA and 3 µl SensiFAST™ SYBR^®^ No-ROX Kit (Bioline) per reaction. Samples were amplified following the manufacturer’s instructions and fluorescence was monitored with a CFX384 Touch™ Real-Time PCR Detection System (Bio-Rad Laboratories, California, USA).

### cDNA synthesis and miRNA expression quantification

For quantification of miRNA expression, low molecular weight cDNA was synthesised by ligation-mediated reverse transcription from 100 to 200 ng of total RNA using a miScript Plant RT Kit (Qiagen, The Netherlands) kit per manufacturer’s protocol. The prepared cDNA was then diluted with milliQ water as per the manufacturer’s instructions for optimal amplification. The qRT-PCR reactions were performed as per manufacturer’s instructions (miScript SYBR Green PCR Kit, Qiagen, The Netherlands) using: 10 µl of 1 × QuantiTect SYBR^®^ green mastermix, 2 µl of 1 × miScript universal primer, 2 µl 0.8 μM miRNA specific primer and 2 µl template cDNA. The qRT-PCR run was performed, and fluorescence was measured using a Rotor-Gene Q 6000 (Qiagen, The Netherlands).

### RNA-Seq library preparation and sequencing

RNA libraries of pooled avocado, macadamia and mango stem, leaf, root, flower and axillary bud tissues were prepared as described by Kerr et al. [23]. cDNA libraries were then normalised using the Trimmer-2 cDNA normalisation kit following the manufacturer’s instructions (Evrogen JSC, Moscow, Russia). Libraries were quantified by qRT-PCR using the Library Quantification Kit for Illumina sequencing platforms (KAPA Biosystems, Boston, USA), using a CFX96 Touch™ Real-Time PCR Detection System (Bio-Rad Laboratories, California, USA). The libraries were normalised to a working concentration of 4 nM using the molarity calculated from qRT-PCR, adjusted for fragment size. The RNA-Seq libraires were then sequenced using an Illumina Sequencer (NextSeq).

### RNA-Seq read quality control assessment and mapping

Trimmomtatic v. 0.35 [24] was used to remove adaptors, trim and filter the reads based on quality. Sequence contaminants were removed from the reads using Deconseq v. 0.4.2 [25]. Sequence read quality was checked using FastQC (http://www.bioinformatics.babraham.ac.uk/projects/fastqc/).

To validate our RNA sequencing results, we mapped the reads to published draft transcriptome and genome sequences. Trimmed reads were mapped to published reference transcriptome and genome assemblies using HISAT v.2 [26] using default settings.

## Results

### Phenol/chloroform-free CTAB/PVP/SDS-based RNA extraction

**Fig. 1:**
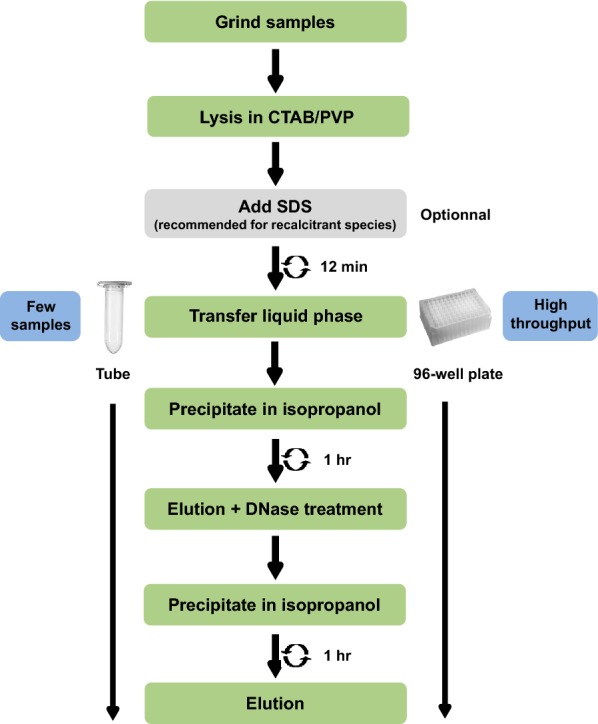
Description of the CTAB/SDS-based RNA extraction procedure

**Fig. 2:**
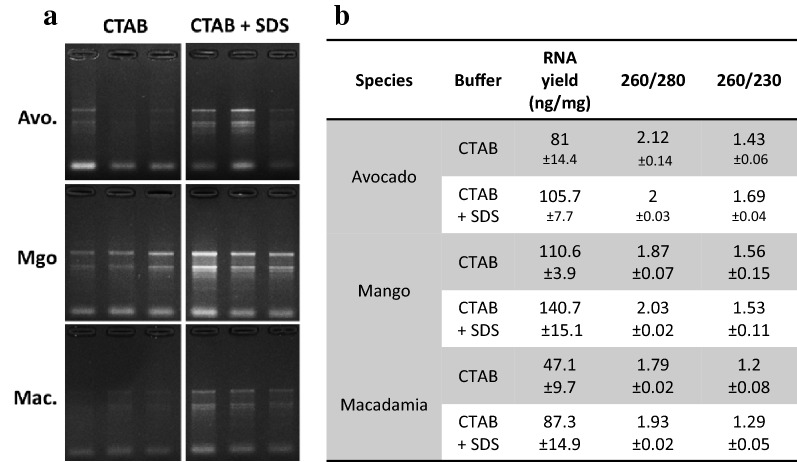
RNA quality control of RNA extraction with the CTAB/SDS method. **a** RNA from avocado, mango and macadamia mature leaf extracted with CTAB (left) or CTAB + SDS (right). 10 µl of the final elution were loaded in each well. **b** Average RNA yield and purity measured on the same samples as A (n = 3)

In the first couple of experiments described below, we developed and tested an RNA extraction method using 40–60 mg of frozen leaf tissue from avocado, macadamia and mango mature leaves. Most of the published RNA extraction procedures developed for recalcitrant tree species improved the RNA yield and quality by using a CTAB/PVP lysis buffer with high concentration of salt (NaCl) as described in the introduction [8, 17]. We therefore used this buffer to lyse our samples. Most protocols use this buffer to extract RNA in conjunction with phenol/chloroform extraction to separate the nucleic acids from the proteins. However, we did not use phenol and chloroform because of their toxicity and also because they do not allow for high throughput implementation in 96-well plates. We therefore decided to precipitate and pellet the RNA by adding a volume of isopropanol and by centrifuging the samples after the lysis step (Fig. 1). At that stage, the proteins and undesirable compounds such as polysaccharides and polyphenols remained solubilised in the supernatant and could be easily removed. After washing the pellet in ethanol, the nucleic acids were resuspended and a DNase treatment applied before another round of precipitation in isopropanol and elution to recover the RNA. The quality control in Fig. 2 shows that the RNA was not degraded and that the ratio 260/280 was above 1.79, suggesting that proteins were removed during the extraction. The 260/230 ratio ranged from 1.2 to 1.56 depending on the species, suggesting that some residual polysaccharides remained.

**Fig. 3:**
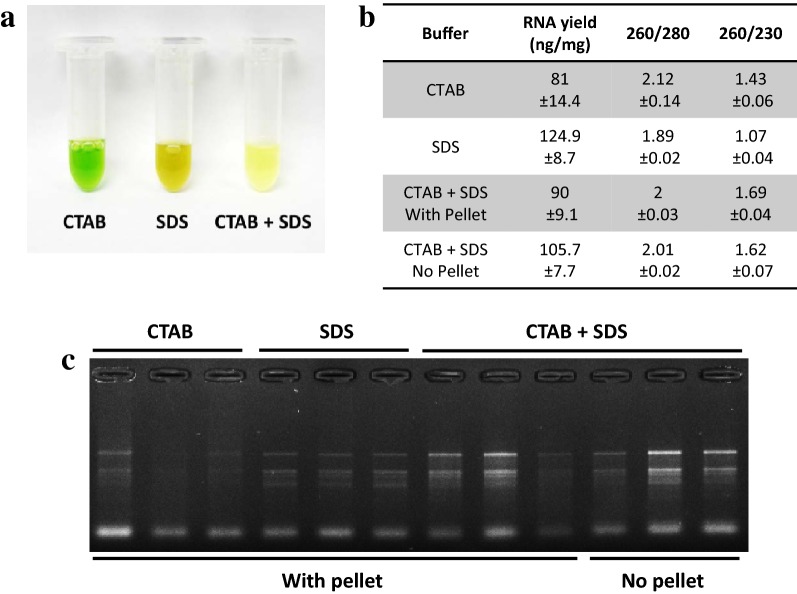
RNA quality control of RNA extraction with the CTAB/SDS method. **a** Picture of avocado leaf samples just after the lysis step with CTAB only, SDS only or CTAB + SDS. **b** Average RNA yield and purity measured on samples extracted with CTAB only, SDS, CTAB + SDS. The CTAB + SDS condition was tested in presence or absence of the debris pellet (respectively referred to as “with pellet” and “no pellet” on the figure). **c** RNA gel of the same samples than B. 10 µl of the final elution were loaded in each well

In order to improve the method, 1% SDS was added to the lysis buffer after the incubation step at 60°. SDS is a detergent which helps digest the cell membrane and release more nucleic acids during this step. SDS also has the property of disrupting the nucleic acid/protein interactions and facilitate the solubilisation of CTAB/nucleic acid complexes [27], leading to improved precipitation of the nucleic acids and increased yields. The RNA yields obtained with samples extracted with SDS by itself gave better results than with the CTAB/PVP buffer. However, the 260/230 ratio was very low (1.07) and oxidation of the samples could not be prevented (Fig. 3a). Combining CTAB and SDS resulted in improved yields (Figs. 2, 3). This was particularly visible with macadamia, for which the RNA yield nearly doubled. As observed by the sample colour after the lysis step (Fig. 3a), the combination of CTAB/PVP and SDS also inhibited sample oxidation and denatured chlorophyll, a known effect of SDS [28]. The interaction between CTAB and SDS was visible through the formation of cloudy precipitate due to the formation of micelles [29, 30] that resulted in the formation of a semi solid white layer at the interface buffer/air after centrifugation. The effect of SDS on yields was not decreased by the absence of CTAB or the presence of the plant tissue in the tube (Fig. 3). This suggests that in this procedure the effect of SDS on yields may be due to its ability to separate proteins from nucleic acids rather than its tissue lysing activity or its property of solubilising the CTAB/nucleic acid complexes. Additionally, adding SDS also slightly increased the 260/230 ratio for avocado and macadamia, suggesting that the combination of CTAB and SDS slightly decreased the amount of polysaccharides in the final elution (Fig. 3).

### RNA extraction from different tissue types

**Fig. 4:**
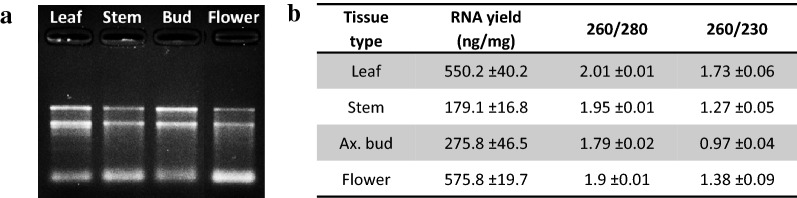
Efficiency the CTAB/SDS method to extract RNA from different tissue types in macadamia. **a** RNA gel from different macadamia tissue types. 500 ng RNA were loaded on a 1.2% agar gel for 20 min. **b** Average RNA yield and purity measured on different tissue samples (n = 4)

We then tested whether the method was efficient with different tissue types. To test this, we performed the same procedure using 15–30 mg of four different macadamia tissue types (leaf, stem, flower, axillary bud). The quality/quantity control demonstrated that the method was efficient on these four tissue types from macadamia (Fig. 4). Root tissues were also tested but gave too inconsistent results to be considered successful (data not included). The amount of RNA extracted from stems and dormant axillary buds was lower than from leaf and flower, probably due to the smaller number of living cells contained in this kind of tissue. For all tissues, the 260/280 was higher than 1.79, showing that proteins had been efficiently removed from the sample. The 260/230 ratio was lower, probably due to the high number of secondary compounds contained in these tissue types.

### RNA extraction using a 96-well plate

**Fig. 5:**
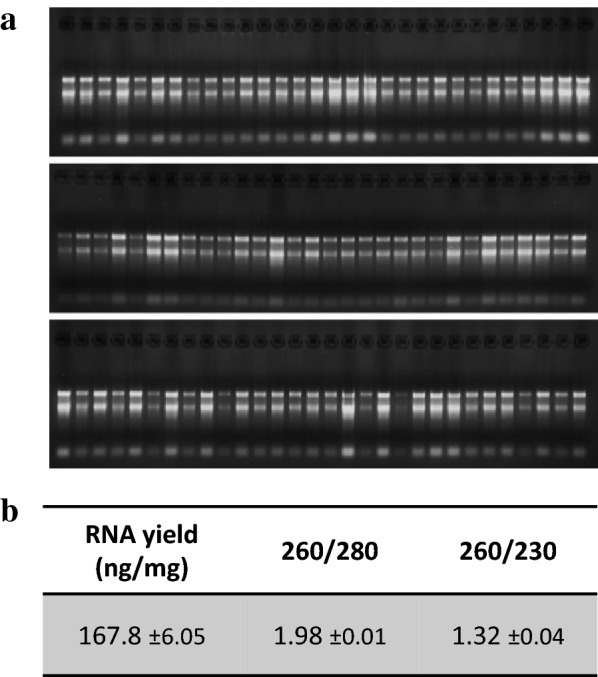
Efficiency of the CTAB/SDS method to extract RNA 96 well plate. **a** RNA from 90 mango leaf samples extracted on a 96 well plate. 8 µl of the final elution were loaded in each well. **b** Average RNA yield and purity measured on the same samples as A (n = 90; ± se)

We wanted to develop the method in a way that allowed us to extract a high number of samples at the same time. To achieve this goal, we took advantage of the fact that after the lysis step, the method does not require pipetting under a fume hood with great precaution as is the case with methods using phenol and chloroform (different phases). We therefore performed the following steps in a 2 ml 96-well plate using the same procedure as with tubes. As with tubes, the extraction performed with plates generated satisfactory results as demonstrated by the quality control shown in the Fig. 5. Using the 96-well plate method allowed the extraction of 96 samples in only one day (grinding time not included) and the time taken was even shorter when using a 96-channel pipette (PLATEMASTER, Gilson).

### Quantitative real-time PCR and RNA-Seq read quality control

**Fig. 6:**
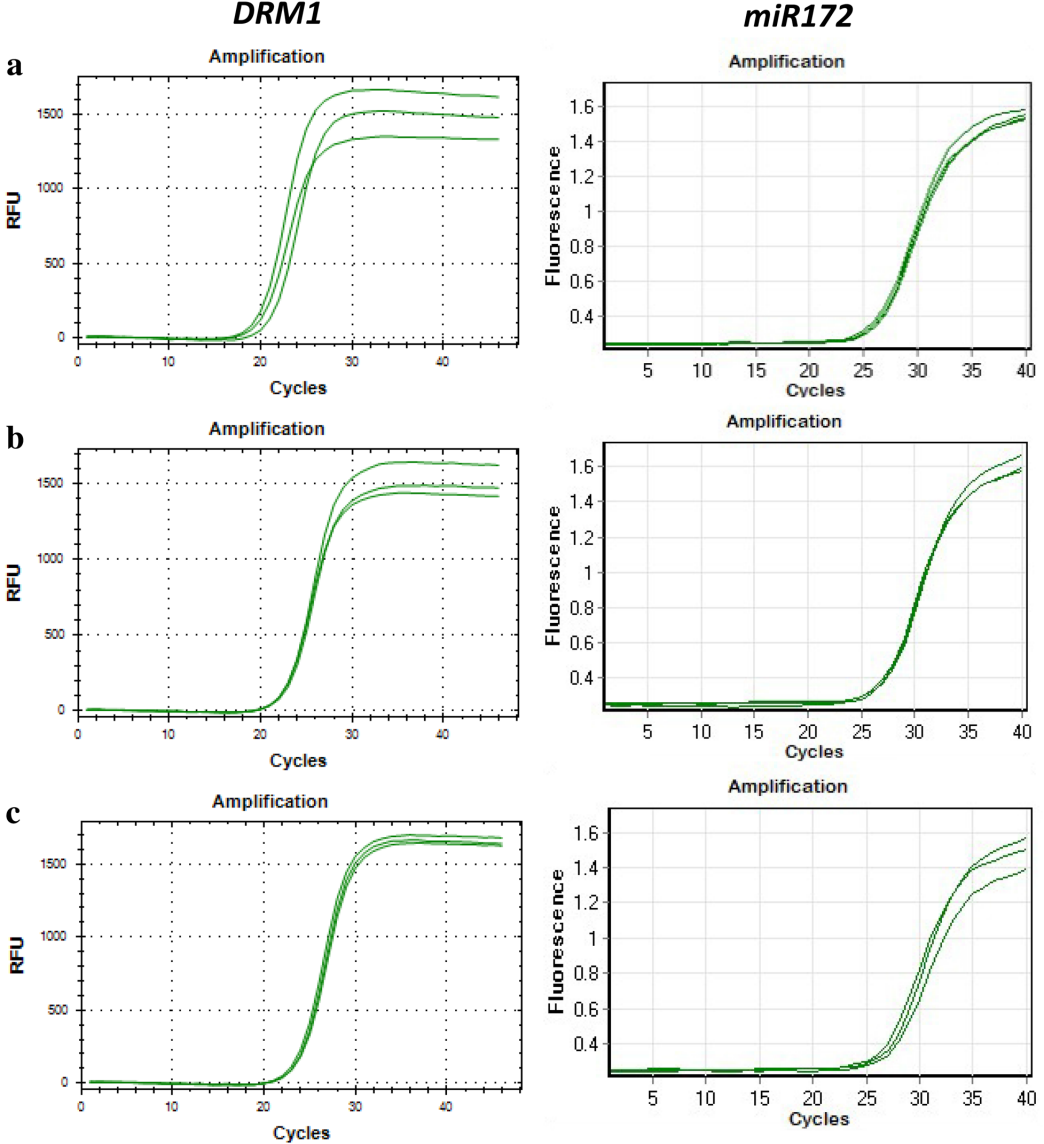
Amplification curves for *DRM1* and *miR172* in avocado (**a**), mango (**b**) and macadamia (**c**). qRT-PCR reactions were performed using the RNA extracted by the CTAB/SDS-based method from the samples shown in Fig. 2 (n = 3)

To demonstrate that the extraction method generates RNA of good enough quality, we used RNA for quantitative real time PCR and RNA sequencing. The amplification (Fig. 6) and melting curves (Additional file 1: Fig. S1) obtained with *DORMANCY1 (DRM1)* and *miR172* in the three tree species demonstrates that the RNA can be used successfully for quantitative RT-PCR (Fig. 6). These results demonstrate that, even though the 260/230 ratio was not optimal in some samples, the quality of the RNA isolated with this method was good enough to perform gene and miRNA quantification.

**Fig. 7:**
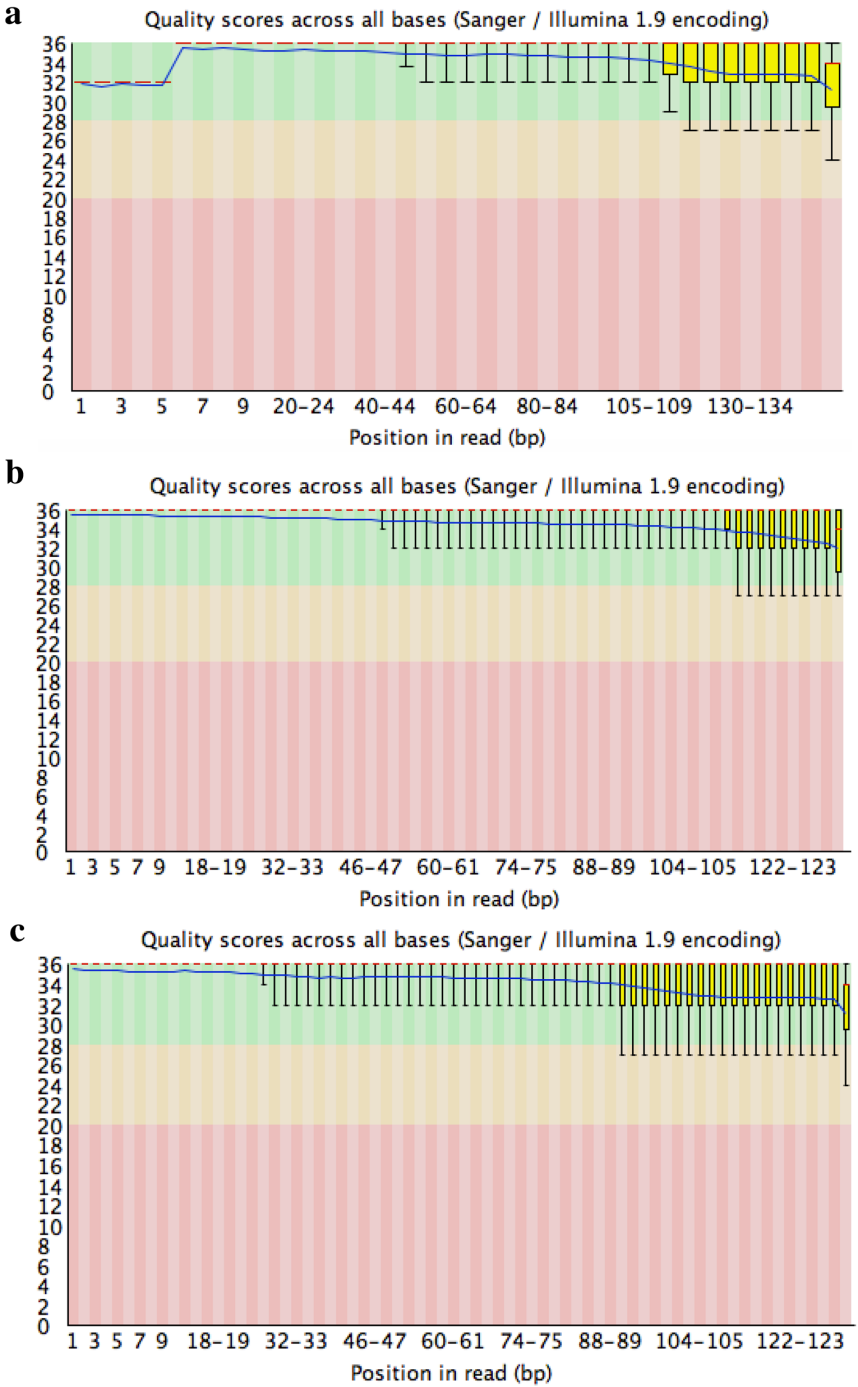
Sequence quality of RNA extracted using the CTAB/SDS RNA extraction protocol. RNA was assessed by producing high-throughput RNA sequencing (RNA-seq) libraries of the avocado, macadamia and mango samples shown in Figs. 1, 2, 3, 4 and 5. The RNA-Seq libraries were pair-end sequenced (150 bp) on the Illumina 2500 Hi-Seq Platform. Sequencing quality assessment using FastQC version 0.10.1 [9] is represented in graphs describing quality across all bases from every sequence read at each position (**a** Avocado; **b** Mango; **c** Macadamia, respectively). Sequence quality is based on *phred* scores, an exponential scale where, for example, 20 = one incorrect sequence base-call in 100, and 30 = one incorrect base-call in 1000. The y-axis shows the quality scores, and the higher the score, the greater confidence in the base-calls at that position. The background of the graph divides the y-axis into very good quality calls (green), reasonable quality (orange), and poor quality (red). The graphs are representative of the forward reads (for reverse reads, see Additional file 2: Fig. S2)

To further demonstrate the efficacy of our RNA extraction protocol, we used it to prepare normalised RNA-Seq libraries for each of avocado, macadamia and mango tree species and sequenced them using Illumina sequencing technology. The quality control of the libraries obtained using this method demonstrates that the RNA extracted with the method described in the paper could be successfully used for such a purpose (Fig. 7 and Additional file 2: Fig. S2). Using FastQC version 0.11.5 (http://www.bioinformatics.babraham.ac.uk/projects/fastqc/) for quality assessment after standard trimming and quality control using Trimmomatic [24], the data indicate good phred quality (Fig. 7) with uniform high quality base calls to the end of the forward (Fig. 7) and reverse (Additional file 2: Fig. S2) sequence read. This indicates most sequence reads were high quality, which provides confidence in downstream genetic analysis.

### Read mapping

To verify our RNA sequencing, we mapped our reads after trimming to published transcriptome and genome assemblies using HISAT v.2 [26] using default settings (Table 1). 76 and 80% of our avocado reads mapped to the *Persea americana* var ‘drymifolia’ [31] and cv. ‘Lisa’ [32] transcriptome assemblies respectively. 66 and 69% of our mango reads mapped to the *Mangifera indica* L. cv. ‘Shelly’ [33] and ‘Zill’ [34] transcriptome assemblies respectively. 79% of our macadamia reads mapped to the *Macadamia integrifolia* cv. 741 ‘Mauka’ draft genome assembly [35]. This quality control further supports that the quality of the RNA extracted with our method is sufficient for a variety of transcriptomic studies.

**Table 1:** List of RNA-seq samples and the percentage and number of mapped reads to different reference transcriptomes

Species	Number of reads	Number of mapped reads	% of reads mapped	NCBI Project No.	Contigs intranscriptome or genome	References
Avocado	160,082,107	122,046,598	76.24	PRJNA282441	83,650	Ibarra-Laclette et al. [31]
Avocado	160,082,107	128,289,801	80.14	PRJNA391003	151,545	Liu et al. [32]
Mango	197,535,231	122,456,877	65.85	PRJNA227243	57,544	Luria et al. [33]
Mango	197,535,231	136,367,200	69.03	PRJNA234455	54,207	Wu et al. [34]
Macadamia	159,049,565	126,873,838	79.77	PRJEB13765	23,452	Nock et al. [35]

### Phenol/chloroform-free CTAB/PVP/SDS-based DNA extraction

**Fig. 8:**
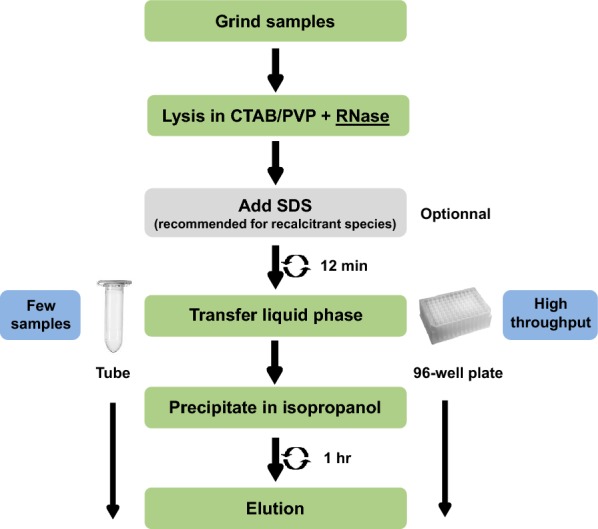
Description of the CTAB/SDS-based DNA extraction procedure

**Fig. 9:**
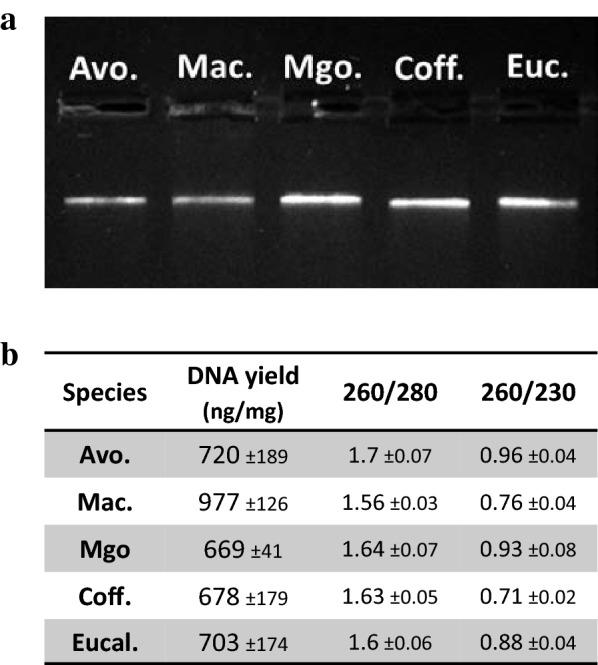
Genomic DNA extracted with the CTAB/SDS method. Genomic DNA was extracted from 20 to 35 mg of mature leaf tissue from avocado, macadamia, mango, coffee and eucalyptus trees using a modified version of the procedure. **a** The quality was assessed by electrophoresis (500 ng DNA loaded on a gel). **b** Quantity and purity control obtained on tree replicates ± standard error

The issues usually met with when performing DNA extraction methods for tree species are the same as with RNA extraction methods. We therefore decided to test whether this RNA extraction method could be converted to a genomic DNA extraction method. To achieve this, we modified the method by replacing the DTT from the CTAB buffer with RNase in order to remove the RNA during the lysis step. As for RNA extraction, 1% SDS was added at the end of the lysis step and a single precipitation/washing/elution round was performed to recover the purified DNA (Fig. 8). We also included mature leaf samples from outdoor grown coffee tree and eucalyptus, two species known to be challenging in terms of DNA extraction [9]. The quality and quantity control results presented in Fig. 9 show that the yields obtained are satisfactory and that the DNA extracted was not degraded, thus demonstrating that this method could be successfully converted into a DNA extraction method for recalcitrant tropical and subtropical species.

## Discussion

### A safer version of the CTAB/PVP-based extraction methods

Successful nucleic acid extraction from species recalcitrant in molecular biology requires the use of CTAB/PVP-based buffers. The results we described here clear demonstrated that this buffer was efficient to extract RNA and DNA from different tropical and subtropical species using challenging for this type of experiment. We also highlight that SDS-based buffer, used in some procedures [36, 37], cannot subsidise the CTAB/PVP buffer without leading to an undesirable browning of the tissue (oxidation) companied with low 260/230 ratio.

Although the CTAB/PVP-based methods are giving good results in term of RNA yield and quality, these methods not compatible with the increasing concern for health safety in the scientific community, due to the large amounts of phenol and chloroform used in these methods. Our results show that it is possible to precipitate RNA directly after the lysis step. However, RNA yields are low, especially for macadamia, a crop underrepresented in molecular studies. Although, CTAB and SDS react together to form micelles, adding 1% SDS at the end of the lysing step with a CTAB/PVP-based buffer could increase the RNA yields. This increase in final RNA yields was likely to be due to the property of SDS to separate proteins from RNA, since the effect of SDS on yield did not rely on the presence of CTAB or plant tissue in the reaction (Fig. 3).

### High recovery yields with small amounts of tissue

In this experiment described in the Fig. 4, the RNA yields for leaves from recalcitrant species were higher than in the experiment described in Fig. 2 and were quite comparable to the yields extracted from Arabidopsis or pea leaves (Additional file 3: Fig. S3). Additionally, the ratio 260/230 was also improved compared to the experiment in Fig. 2. These differences may be explained by the different amounts of fresh tissues which were used in these two experiments and suggest that this method is more efficient when performed with less fresh tissue. Interestingly, the results obtained on single pea buds (less than 0.5 mg) demonstrate that RNA recovery is very high (Additional file 3: Fig. S3). This suggests that this procedure gives better results with small amounts of tissue (less than 25 mg) and that this method can be used to recover high RNA yield, which is an advantage for samples for which on small amount of tissue can be collected.

### Facilitating molecular studies in orphan crops

Avocado, macadamia and mango can be classified as orphan crops as they are agriculturally important yet they are not well studied and therefore have limited genetic and genomic resources [38]. The impracticality and unsafety of the published nucleic acid extraction methods working with these crops is likely to contribute to this effect. The methods we developed in this study demonstrate that this simple and safer procedure allows to extract quality RNA for qRT-PCR from mRNA and also microRNA which are of emerging importance in molecular plant biology [7]. Moreover, the method described here is compatible with sequencing technology. This aspect is important since transcriptomic analysis are now routinely performed in laboratories. Our method will hopefully contribute to bridging the gap between model crops and these orphan crops.

## Conclusion

Within the last decade, the price of transcriptome sequencing has decreased considerably, prompting researchers to design larger scaled experiments. However, the toxicity and impracticality of the chemicals used for nucleic acids extraction for woody tropical species represent a major issue for these large-scale experiments. The method we described here constitutes a solution to alleviate these issues and will prompt for more large-scale experiments led on species considered recalcitrant in molecular biology.

## Additional files

**Additional file 1: Fig. S1.** Melting curves for *DRM1* and *miR172* in avocado (A), mango (B) and macadamia (C). qRT-PCR reactions were performed using the RNA extracted by the CTAB/SDS-based method from the samples shown in Figs. 2 and 6 (n = 3).

**Additional file 2: Fig. S2.** Sequence quality of RNA extracted using the CTAB/SDS RNA extraction protocol. RNA was assessed by producing high-throughput RNA sequencing (RNA-seq) libraries of the avocado, macadamia and mango samples shown in Figs. 1, 2, 3, 4 and 5. The RNA-Seq libraries were pair-end sequenced (150 bp) on the Illumina 2500 Hi-Seq Platform. Sequencing quality assessment using FastQC version 0.10.1 [9] is represented in graphs describing quality across all bases from every sequence read at each position (**A-Avocado; B-Mango; C-Macadamia**, respectively). Sequence quality is based on *phred* scores, an exponential scale where, for example, 20 = one incorrect sequence base-call in 100, and 30 = one incorrect base-call in 1000. The y-axis shows the quality scores, and the higher the score, the greater confidence in the base-calls at that position. The background of the graph divides the y-axis into very good quality calls (green), reasonable quality (orange), and poor quality (red). The graphs are representative of the reverse reads (for forward reads, see Fig. 7).

**Additional file 3: Fig. S3.** Efficiency the CTAB/SDS method to extract RNA from different tissue types in Arabidopsis and pea. Average RNA yield and purity measured on different tissue samples (data are average ± standard error; n = 3–4, or 8 for single buds). Single pea buds are individual dormant axillary buds (less than 0.5 mg each).

## Data Availability

Not applicable.

## Abbreviations

CTAB: cetyltrimethylammonium bromide
PVP: polyvinylpyrrolidone
SDS: sodium dodecyl sulfate
DTT: dithiothreitol
EDTA: ethylenediaminetetraacetic acid
