# Supplementary figures and images for: A phenol/chloroform-free method to extract nucleic acids from recalcitrant, woody tropical species for gene expression and sequencing
Source: Plant Methods. 2019 Jun 5;15:62. doi: 10.1186/s13007-019-0447-3 (PMC6549277; doi:10.1186/s13007-019-0447-3)

## Slide 1
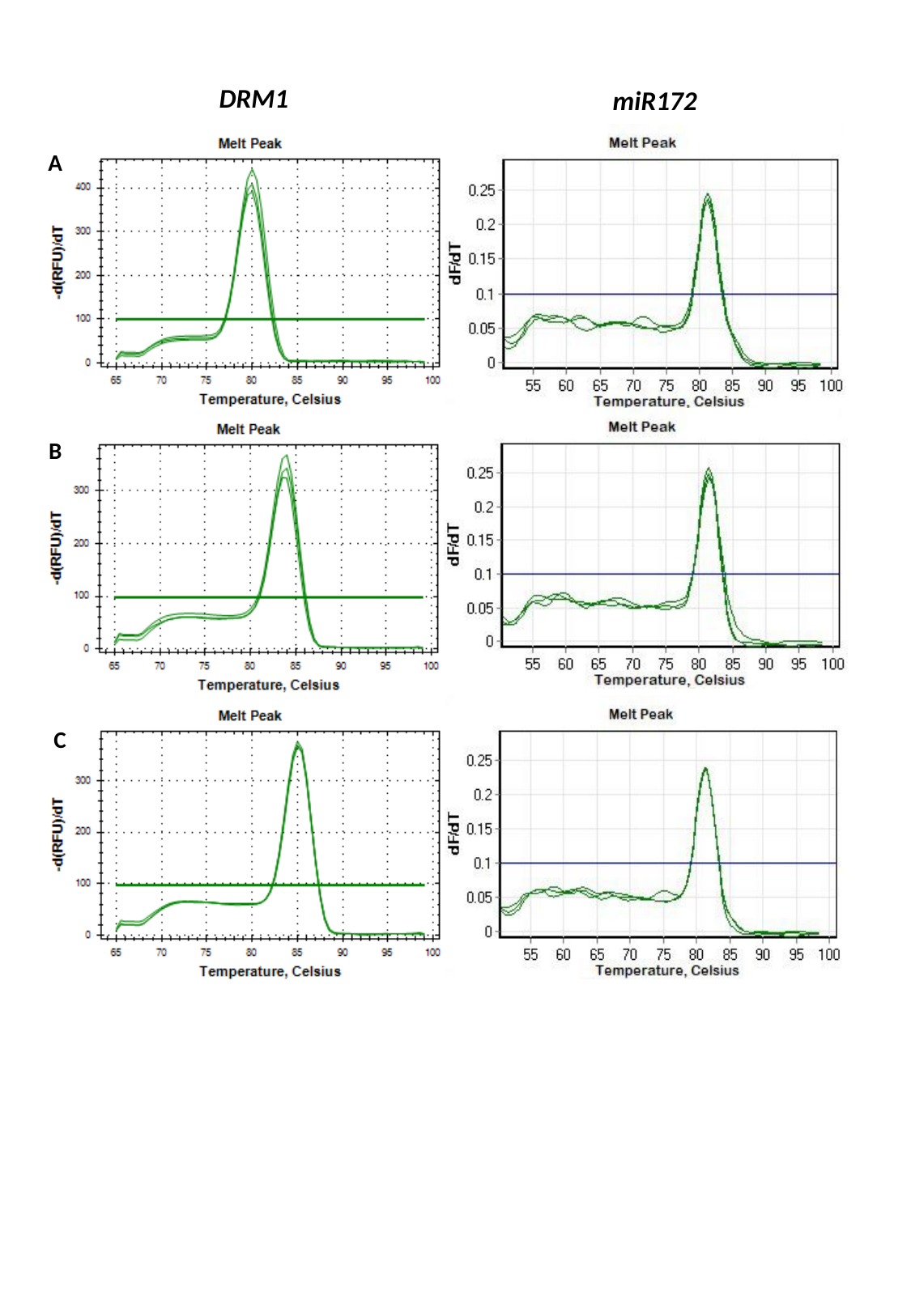

DRM1
miR172
A
B
C

Supplement: Supplementary file 1 — Additional file 1: Fig. S1. Melting curves for DRM1 and miR172 in avocado (A), mango (B) and macadamia (C). qRT-PCR reactions were performed using the RNA extracted by the CTAB/SDS-based method from the samples shown in Figs. 2 and 6 (n = 3). [file 13007_2019_447_MOESM1_ESM.pptx]

## Slide 1
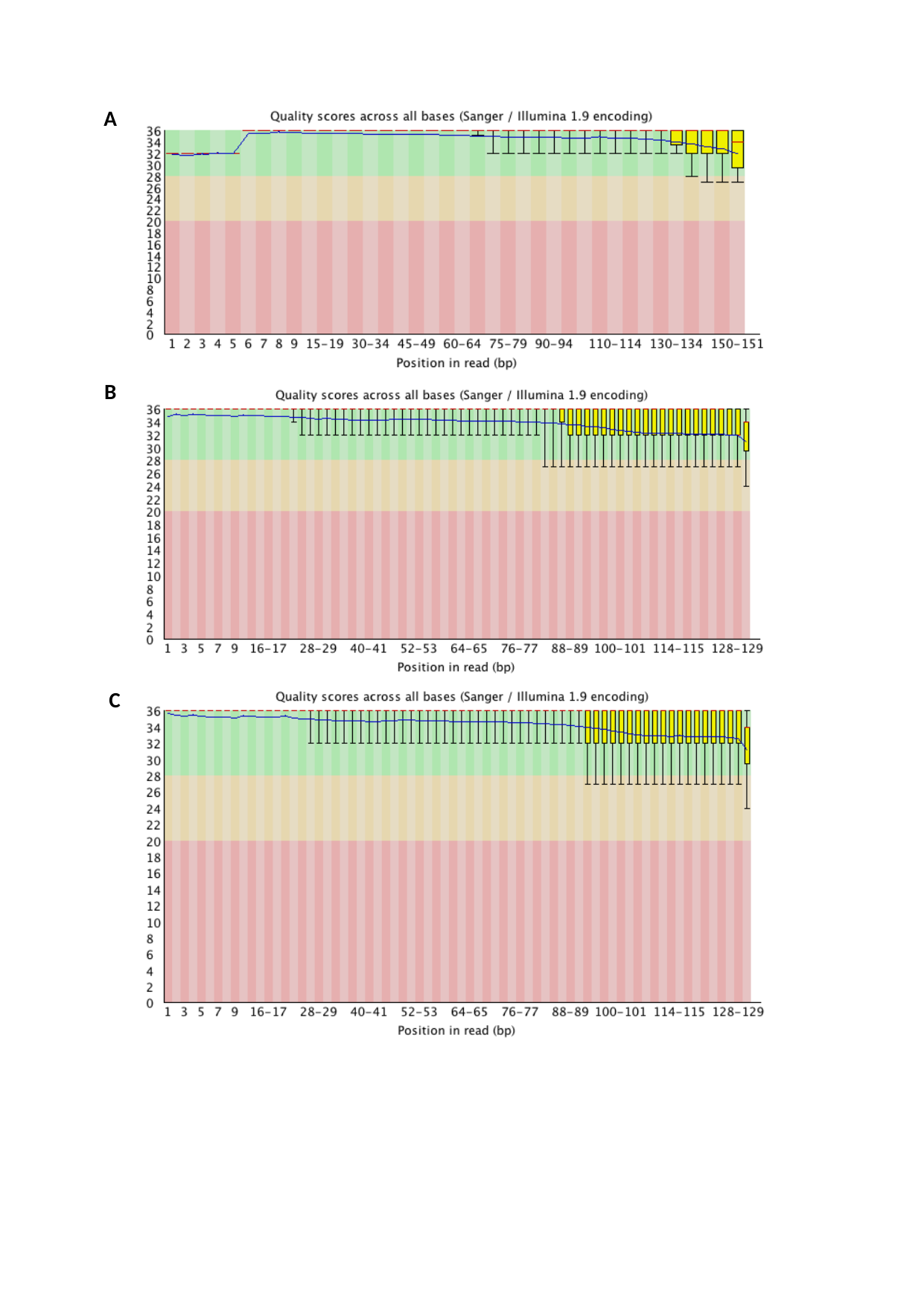

A
B
C

Supplement: Supplementary file 2 — Additional file 2: Fig. S2. Sequence quality of RNA extracted using the CTAB/SDS RNA extraction protocol. RNA was assessed by producing high-throughput RNA sequencing (RNA-seq) libraries of the avocado, macadamia and mango samples shown in Figs. 1, 2, 3, 4 and 5. The RNA-Seq libraries were pair-end sequenced (150 bp) on the Illumina 2500 Hi-Seq Platform. Sequencing quality assessment using FastQC version 0.10.1 [9] is represented in graphs describing quality across all bases from every sequence read at each position (A-Avocado; B-Mango; C-Macadamia, respectively). Sequence quality is based on phred scores, an exponential scale where, for example, 20 = one incorrect sequence base-call in 100, and 30 = one incorrect base-call in 1000. The y-axis shows the quality scores, and the higher the score, the greater confidence in the base-calls at that position. The background of the graph divides the y-axis into very good quality calls (green), reasonable quality (orange), and poor quality (red). The graphs are representative of the reverse reads (for forward reads, see Fig. 7). [file 13007_2019_447_MOESM2_ESM.pptx]
