# Supplementary material for: A phenol/chloroform-free method to extract nucleic acids from recalcitrant, woody tropical species for gene expression and sequencing
Source: Plant Methods. 2019 Jun 5;15:62. doi: 10.1186/s13007-019-0447-3 (PMC6549277; doi:10.1186/s13007-019-0447-3)
Supplement: Supplementary file 3 — Additional file 3: Fig. S3. Efficiency the CTAB/SDS method to extract RNA from different tissue types in Arabidopsis and pea. Average RNA yield and purity measured on different tissue samples (data are average ± standard error; n = 3–4, or 8 for single buds). Single pea buds are individual dormant axillary buds (less than 0.5 mg each). [file 13007_2019_447_MOESM3_ESM.pptx]

## Slide 1
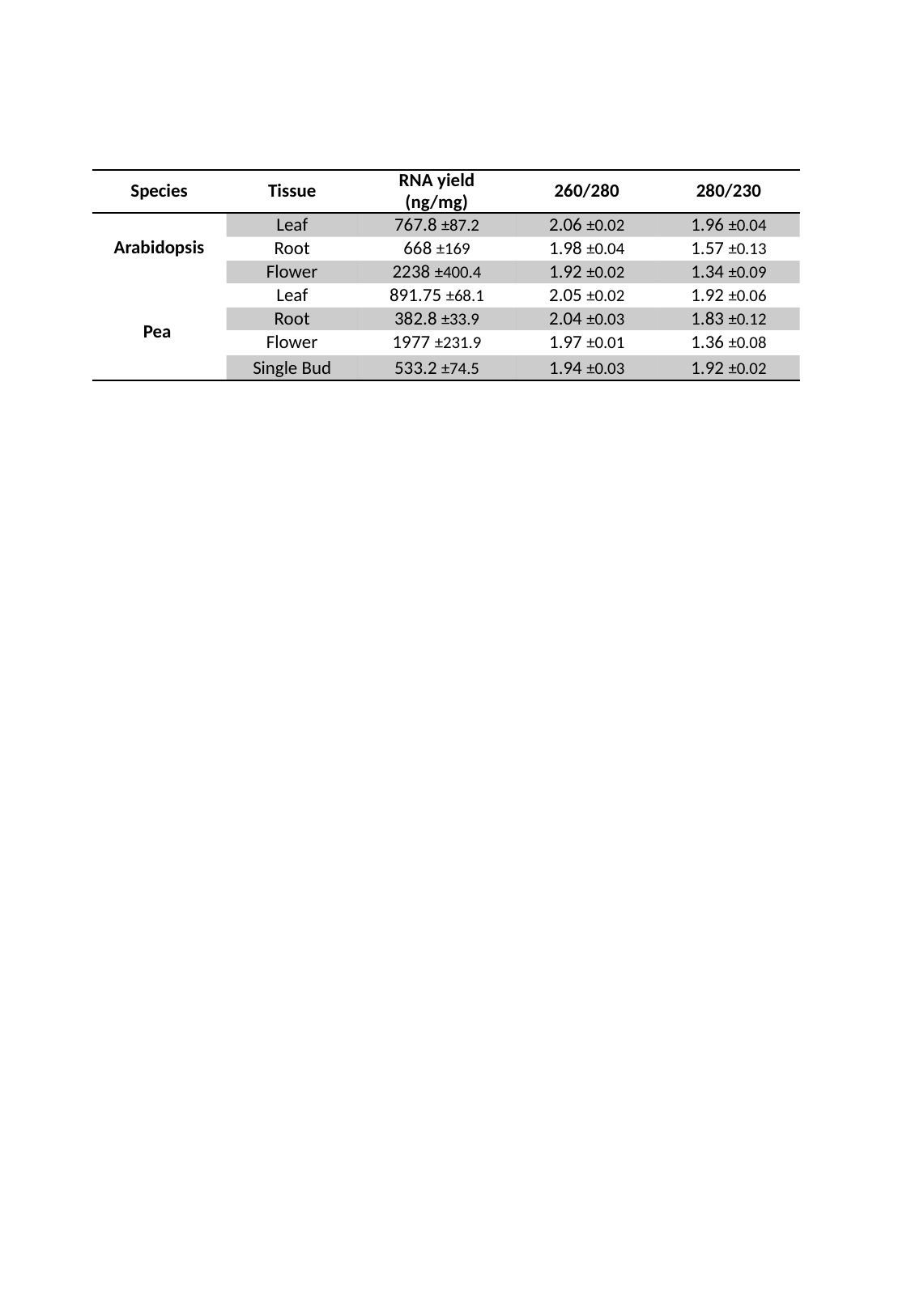

| Species | Tissue | RNA yield (ng/mg) | 260/280 | 280/230 |
| --- | --- | --- | --- | --- |
| Arabidopsis | Leaf | 767.8 ±87.2 | 2.06 ±0.02 | 1.96 ±0.04 |
| | Root | 668 ±169 | 1.98 ±0.04 | 1.57 ±0.13 |
| | Flower | 2238 ±400.4 | 1.92 ±0.02 | 1.34 ±0.09 |
| Pea | Leaf | 891.75 ±68.1 | 2.05 ±0.02 | 1.92 ±0.06 |
| | Root | 382.8 ±33.9 | 2.04 ±0.03 | 1.83 ±0.12 |
| | Flower | 1977 ±231.9 | 1.97 ±0.01 | 1.36 ±0.08 |
| | Single Bud | 533.2 ±74.5 | 1.94 ±0.03 | 1.92 ±0.02 |
